# Anterior encephalocele: a series of 15 patients considering the surgical points and survival

**Published:** 2012-11

**Authors:** Farideh Nejat

**Affiliations:** ^a^Children Hospital Medical Center, Tehran University of Medical Sciences, Tehran, Iran.

## Abstract

**Background:**

Anterior encephalocele is a rare pathologic condition in pediatric neurosurgery. Most of encephaloceles are located in the occipital region. The present study reports a series of 15 children with anterior encephaloceles.

**Methods:**

This was a descriptive retrospective survey conducted on the medical recordings of encephalocele patients underwent a neurosurgery during a 12-year period. A total of 83 cases of neurosurgery performed in a pediatric hospital (Children Hospital Medical Center, Tehran, Iran) were reviewed. The demographic, surgical approach and follow up data and recordings of these patients were collected and then analyzed.

**Results:**

A total of 83 patients with encephalocele were operated, of which 15 have anterior encephalocele. They were classified as fronto-orbital, frontonasal, frontoethmoidal, transsphenoidal, and interfrontal. They were aged from 3 days to 7 years at the time of operation. All surgeries were performed with craniotomy or endoscopic repair of the lesion. A 1 to12-year period follow-up showed that all cases are alive and all of them except for one case have normal neurodevelopmental status.

**Conclusions:**

Anterior encephalocele is a rare condition in congenital central nervous system. Most of patients with anterior encephalocele encounter with face deformity because of mass or hypertelorism or orbital disfiguration. Some of them have serious complications of meningitis due to CSF leakage. In time surgical management with precious correction of cranial bone defect are necessary to prevent more complicated consequences and to reduce the need for surgery in the future.

**Keywords:**

Anterior encephalocele, surgery, skull defect


					Volume 4, Suppl. 1 Nov 2012
Publisher: Kermanshah University of Medical Sciences
URL: http://www.jivresearch.org
Copyright:
Congress of Iranian Neurosurgeons, 14-16 November 2012, Kermanshah, Iran
Paper No. 14
Anterior encephalocele: a series of 15 patients considering the
surgical points and survival
Farideh Nejat a,*
a
Children Hospital Medical Center, Tehran University of Medical Sciences, Tehran, Iran.
Abstract:
Background: Anterior encephalocele is a rare pathologic condition in pediatric neurosurgery. Most of encephaloceles
are located in the occipital region. The present study reports a series of 15 children with anterior encephaloceles.
Methods: This was a descriptive retrospective survey conducted on the medical recordings of encephalocele patients
underwent a neurosurgery during a 12-year period. A total of 83 cases of neurosurgery performed in a pediatric
hospital (Children Hospital Medical Center, Tehran, Iran) were reviewed. The demographic, surgical approach and
follow up data and recordings of these patients were collected and then analyzed.
Results: A total of 83 patients with encephalocele were operated, of which 15 have anterior encephalocele. They
were classified as fronto-orbital, frontonasal, frontoethmoidal, transsphenoidal, and interfrontal. They were aged
from 3 days to 7 years at the time of operation. All surgeries were performed with craniotomy or endoscopic repair
of the lesion. A 1 to12-year period follow-up showed that all cases are alive and all of them except for one case
have normal neurodevelopmental status.
Conclusions: Anterior encephalocele is a rare condition in congenital central nervous system. Most of patients with
anterior encephalocele encounter with face deformity because of mass or hypertelorism or orbital disfiguration.
Some of them have serious complications of meningitis due to CSF leakage. In time surgical management with
precious correction of cranial bone defect are necessary to prevent more complicated consequences and to reduce
the need for surgery in the future.
Keywords:
Anterior encephalocele, surgery, skull defect
* Corresponding Author at:
Farideh Nejat: Associated Professor of Neurosurgery, Children's hospital Medical Center, Tehran University of Medical Sciences, Tehran, Iran,
Tel: 09121494064, Email: nejat@sina.tums.ac.ir, (Nejat F.).

				

